# Extending the Applicability of the Dose Addition Model to the Assessment of Chemical Mixtures of Partial Agonists by Using a Novel Toxic Unit Extrapolation Method

**DOI:** 10.1371/journal.pone.0088808

**Published:** 2014-02-12

**Authors:** Martin Scholze, Elisabete Silva, Andreas Kortenkamp

**Affiliations:** Institute for the Environment, Brunel University, Uxbridge, Middlesex, United Kingdom; Universidad Miguel Hernández de Elche, Spain

## Abstract

Dose addition, a commonly used concept in toxicology for the prediction of chemical mixture effects, cannot readily be applied to mixtures of partial agonists with differing maximal effects. Due to its mathematical features, effect levels that exceed the maximal effect of the least efficacious compound present in the mixture, cannot be calculated. This poses problems when dealing with mixtures likely to be encountered in realistic assessment situations where chemicals often show differing maximal effects. To overcome this limitation, we developed a pragmatic solution that extrapolates the toxic units of partial agonists to effect levels beyond their maximal efficacy. We extrapolated different additivity expectations that reflect theoretically possible extremes and validated this approach with a mixture of 21 estrogenic chemicals in the E-Screen. This assay measures the proliferation of human epithelial breast cancers. We found that the dose-response curves of the estrogenic agents exhibited widely varying shapes, slopes and maximal effects, which made it necessary to extrapolate mixture responses above 14% proliferation. Our toxic unit extrapolation approach predicted all mixture responses accurately. It extends the applicability of dose addition to combinations of agents with differing saturating effects and removes an important bottleneck that has severely hampered the use of dose addition in the past.

## Introduction

Dose addition (DA, here used synonymously with concentration addition) is a widely used pharmacological concept for the prediction of chemical mixture effects when only the toxicity of individual components is known [1], [2]. Various risk assessment methods for evaluating combined exposures are in use (e.g., toxic equivalent factor approach, toxic unit summation, hazard index and the point of departure index), and without exception all these methods are derived from DA [3].For a wide variety of mixtures and toxicological endpoints DA has proven remarkably successful in formulating a dose additivity null hypothesis [4], [5]. This hypothesis expresses the expected combination effect based on the assumption that all mixture components exert their effects without influencing each other's action. Using the DA additivity hypothesis as a point of reference, it is then possible to assess experimentally observed mixture effects in terms of synergism or antagonisms. Still lacking is sufficient empirical evidence about the joint action of environmentally realistic mixtures, composed of agents from different chemical and functional classes. This makes it difficult to validate the assumption that DA might be applicable as a general “rule of thumb” for describing the joint action of chemical mixtures. A crucial prerequisite is that the mathematical features of DA are capable of dealing with these more difficult mixture scenarios.

**Figure 1:**
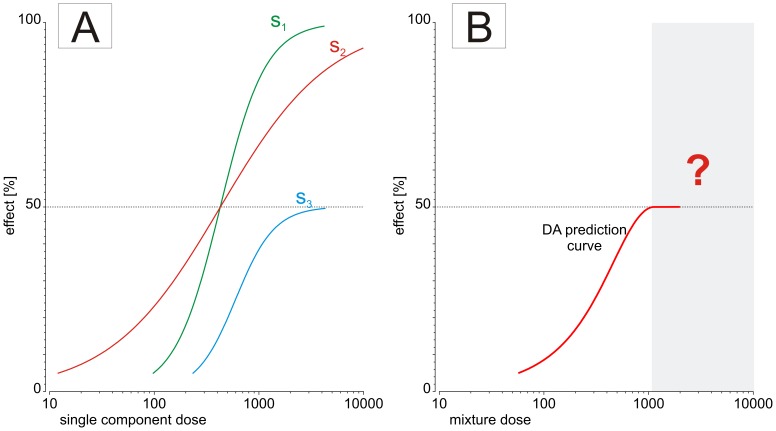
Example of dose-response curves from three mixture components (A) and their joint effect curve predicted by dose addition (B), for serial dilutions of a mixture with fixed mixture ratio proportional to their EC40s.

One such difficulty relates to mixtures composed of chemicals that show differing maximal effects. Due to the mathematical features of DA, the concept cannot be applied to effect levels that exceed the maximal effect of the least efficacious compound present in the mixture. As shown in Figure 1, this limits its usefulness when dealing with mixtures composed of substances that show partial agonism, where the effects at saturating concentrations are somewhat smaller than is biologically achievable. This has been observed with some aryl hydrocarbon receptor (AhR) agonists and certain estrogenic agents [6], [7], [8], [9]. Similar problems occur when dealing with mixtures of chemicals that show hormesis, as seen with phytotoxicants acting on plant species [10].

Several attempts have been made to overcome these difficulties. They all deal with the original mathematical formulation of DA that defines a combination effect: a mixture composed of *n* components with doses *d_1_* of the first component, *d_2_* of the second component, and *d_n_* for the n-th component is dose additive when
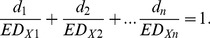
(1)


Here, *ED_X1_*, *ED_X1_* …, *ED_Xn_* are the doses of the individual components that on their own produce the same effect *X* as the mixture [11], [12]. These effect doses for a common effect level have to be derived from regression models that describe the dose-response relationships of all chemicals present in the mixture for the effect of interest. The quotients *d_n_*/*ED_Xn_* are called toxic units. Equation 1 requires knowledge of the dose of each mixture component that on its own produces the effect magnitude under consideration. For this reason, equation 1 cannot be used for predicting mixture effects that exceed the maximal effect of the least efficacious component, because that effect dose cannot be defined.

As can be seen from equation (1), a mixture effect for a pre-defined effect level *X* is described implicitly and can rarely be solved analytically. Only by using an iterative algorithm can the solution be found numerically. However, under certain circumstances it is possible to re-arrange equation (1) into an explicit expression such that the combined effect of the mixture is described as a function of the effects of its components. Consequently, any combination of full and partial agonists should then allow the calculation of their combined effects and therefore overcome the dilemma shown in Figure 1. Unfortunately, this ideal scenario can only be achieved by making certain simplifying assumptions about the regression models used for approximating the dose-response relationships of individual mixture components [13], [14], [15]. For example, Howard et al [14] utilized a simplified version of the Hill function where instead of the usual three parameters only two were used, one for maximal effect, and the other for location (EC_50_). The slope parameter was kept fixed for all chemicals, presumably because the agents under investigation displayed curves of similar steepness. Howard and colleagues termed their solution “Generalized Concentration Addition” (GCA), and it afforded sufficient flexibility for the accurate prediction of AhR-dependent gene expression for full and partial AhR agonists, as well as competitive antagonists [15].

However, the simplifying assumptions that have to be made during the modeling of dose-response relationships for the individual mixture components may impact negatively on the quality of mixture effect predictions under DA, particularly when the biological data for the single chemicals require more complex non-linear regression functions. This is the case with estrogenic agents where dose-response curves with different slopes are common [9], [16]. The most widely used regression functions for dose-response analyses have at least three model parameters, and these are usually needed for the adequate description of global data differences in terms of position (potency), steepness and maximal effect plateaus [17]. But with these non-linear regression functions it is not possible to re-arrange equation (1) into an explicit functional form that would allow for the analysis of partial agonists, and therefore an extension of the CGA model on basis of more flexible dose-response models cannot be achieved.

In view of these difficulties we became interested in exploring alternative quantitative approaches for dealing with mixtures composed of partial agonists. We reasoned that a straightforward pragmatic solution could be found by extrapolating the dose-response curves of partial agonists to higher effect levels beyond their leveling-off range. The extrapolated effects could then be used for the calculation of the dose addition null hypothesis. The advantage of this approach is that it can accommodate quite complex regression models for the dose-response data of the individual mixture components. However, the use of extrapolation methods is fraught with its own difficulties, particularly in relation to the selection of an appropriate slope for the extrapolated dose-response function. Too steep a gradient may overestimate the contribution of the partial agonist to the overall mixture effect in relation to the other mixture components, and consequently bias the dose addition prediction. The challenge in using extrapolation methods lies in ensuring that the dose addition prediction is as close as possible to the experimentally observed dose additive effect. In principle, this can be achieved by providing a range of potential additivity expectations, based on assumptions of the value of the toxic units in equation (1) that reflect theoretically possible extremes (“toxic unit extrapolation”).

We tested a mixture of 21 estrogenic chemicals and compared the accuracy of mixture effect predictions derived from a toxic unit extrapolation approach with those produced by the GCA method. The estrogenic agents included compounds as diverse as steroidal hormones (endogenous and synthetic), pesticides, cosmetic additives and phytoestrogens, all with dose-response curves that exhibited widely varying shapes, slopes and maximal effects ([9], [16]). The accuracy of both predictions was evaluated by comparison with the combination effects observed experimentally with the E-Screen, an assay sensitive to the proliferative effects of estrogen receptor agonists in a human epithelial breast cancer cell line, MCF-7 BOS [18]. Although cell proliferation offers the possibility to study processes that might interfere with steroid receptor signaling through events beyond estrogen receptor (ER) binding (e.g., activation of growth factor signaling cascades), these processes are believed to converge on the activation of the ER, which ultimately is responsible for cell division and proliferation. We consider, therefore, that the pharmacological assumption of DA is fulfilled, i.e. all compounds act on the same toxicological endpoint by a common mechanism of action. Accordingly, we used DA as the mixture assessment concept for the evaluation of our data.

## Materials and Methods

### Chemicals

17β-Estradiol (E2, 99% purity), estrone (99%), aldrin (98.6%), dieldrin (99.8%), endosulfan α (I, 99.5%), endosulfan β (II, 99.2%), methoxychlor (99.5%), *o,p*'-DDT (97.5%), *o,p*'-DDD (99%), *p,p*'-DDT (99.1%), *p,p*'-DDE (99.5%), β-hexachlorocyclohexane (β-HCH, 98.1%), n-butylparaben, n-propylparaben, and bisphenol A (>99%) were purchased from Sigma-Aldrich Company (Dorset, UK). 3-(4-methylbenzylidene)camphor (4-MBC, Eusolex 6300, >99.7%) and octyl-methoxycinnamate (OMC, Eusolex 2292, >98%) were from VWR international (Poole, UK). 3-Benzylidene camphor (3-BC, Unisol-22, >97%) was from Induchem (Volketswil, Switzerland). Genistein was obtained from Alfa Aesar (Lancashire, UK), and 6-acetyl-1,1,2,4,4,7-hexamethyltetraline (AHTN, tonalide) and hexahydrohexamethylcyclopentabenzopyran (HHCB, Galoxolide) from LGC Promochem (Teddington, UK). All chemicals were used as supplied and stock solutions (1 – 10 mM) were prepared in HPLC-grade ethanol (VWR international). Stock solutions and subsequent dilutions were stored at −20°C. All remaining chemicals were purchased from Sigma-Aldrich, unless stated otherwise.

### Routine cell culture

MCF-7 BOS breast cancer cells were kindly provided by Ana Soto (Tufts University, Boston), who cloned the cells (C7MCF-7) from the original MCF-7 cells obtained from the Michigan Cancer Foundation [18]. Cells were routinely maintained in 75 cm^2^ canted-neck tissue culture flasks in Dulbecco's modified Eagle's medium (DMEM, Invitrogen Corporations, U.K.) supplemented with 5% fetal bovine serum (FBS, Invitrogen) and 1% (v/v) MEM nonessential amino acids (MEM-NEAA, Invitrogen) in a humidified incubator, at 37°C, with 5% CO_2_. Cells were subcultured at approximately 70% confluence over a maximum of 10 passages and regularly tested for Mycoplasma contamination.

### E-Screen assay procedure

The protocol described previously [9], carried out in 96-well micro-titer plates, was used. A detailed description of the data normalisation procedure that we employed can be found in [7]. All components and the mixture were tested in at least four independent experiments, run on up to three micro-titer plates, with each plate containing eight increasing concentrations of the test chemical in duplicates. Some of the data for the single components have been published in [16]. To ensure that none of the 21 compounds dominated the overall mixture effect, they were combined in proportion to their EC_10_ values, concentrations associated with 10% of the maximal effect achievable with saturating concentrations of 17β-estradiol. This effect level was the highest common level for all compounds that we could determine with high statistical certainty, and which was well above the statistical detection limit of the assay. The exact composition of the mixture analysed is given in Table 1.

**Table 1:** Estrogenicity of individual compounds and mixture.

Substance	regression model						EC_10_		Relative proportions (percentages)
*(by order of EC_10_)*	RM	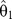	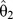	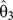	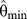	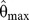	nM [CI]		in test mixture
17β-estradiol	Logit	3.32	1.76	--	0*	0.99	7.56E-04	[5.63E-04;1.02E-03]	1.493E-06
Estrone	Glogit I	0.50	2.59	0.8	0*	1.12	4.61E-02	[3.76E-02;6.09E-02]	1.081E-04
Genistein	Logit	−6.56	3.25	--	0*	0.84	2.52E+01	[1.90E+01;4.40E+01]	5.907E-02
Bisphenol A	Logit	−7.23	2.72	--	0*	0.92	7.61E+01	[5.64E+01;1.00E+02]	1.784E-01
*o,p*'-DDT	Weibull	−6.62	2.37	--	0*	0.77	9.04E+01	[6.48E+01;1.50E+02]	2.119E-01
Butyl paraben	Logit	−8.77	2.53	--	0*	0.88	4.28E+02	[3.25E+02;5.92E+02]	1.003E-00
Endosulfan α (I)	Weibull	−8.36	2.40	--	0*	0.79	4.49E+02	[3.18E+02;6.67E+02]	1.053E-00
β-HCH	Weibull	−10.51	3.13	--	0*	0.85	4.98E+02	[3.63E+02;6.01E+02]	1.167E-00
3-BC (Unisol S-22)	Probit	−6.62	2.02	--	0*	0.73	5.39E+02	[4.50E+02;7.04E+02]	1.263E-00
*o,p*'-DDD	Glogit I	−15.02	4.56	0.8	0*	0.68	6.11E+02	[4.75E+02;8.41E+02]	1.432E-00
Endosulfan β (II)	Weibull	−10.82	3.19	--	0*	0.61	7.52E+02	[6.08E+02;9.40E+02]	1.763E-00
Methoxychlor	Glogit I	−16.34	4.72	0.6	0*	0.50	8.09E+02	[5.78E+02;1.23E+03]	1.896E-00
Propyl paraben	Logit	−12.54	3.61	--	0*	0.86	8.13E+02	[6.95E+02;8.88E+02]	1.905E-00
4-MBC (Eusolex 6300)	Weibull	−10.12	2.79	--	0*	0.42	1.45E+03	[1.27E+03;2.15E+03]	3.399E-00
*p,p*'-DDT	Glogit I	−17.27	4.70	0.7	0*	0.50	1.63E+03	[1.31E+03;1.95E+03]	3.816E-00
Dieldrin	Weibull	−12.16	3.49	--	0*	0.27	1.80E+03	[1.25E+03;2.72E+03]	4.212E-00
*AHTN (Tonalide)*	Weibull	−9.90	2.56	--	0*	0.35	2.74E+03	[2.08E+03;3.57E+03]	6.444E-00
OMC (Eusolex 2292)	Glogit I	−4.30	1.69	6.4	0*	0.29	3.47E+03	[1.89E+03;7.57E+03]	8.143E-00
*p,p*'-DDE	Glogit I	−13.58	4.05	4.1	0*	0.41	3.71E+03	[3.42E+03;4.94E+03]	8.694E-00
Aldrin	Glogit I	−27.81	6.76	0.4	0*	0.20	7.64E+03	[6.21E+03;1.14E+04]	1.791E+01
*HHCB (Galaxolide)*	Weibull	−7.95	1.96	--	0*	0.14	1.51E+04	[1.25E+04;2.00E+04]	3.545E+01
Mixture of 21 components	Weibull	−11.64	3.02	--	0*	0.57	2.04E+03	[1.75E+03; 2.44E+03]	

EC10: concentration associated with 10% proliferation rate. Values in brackets denote the upper and lower limits of the approximate 95% confidence interval based on bootstrap replicates; the column “RM” indicates the mathematical regression function, used for describing the concentration response relationships (see [*17*] for more details). 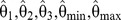 estimated model parameters, if marked by *, then held fixed, i.e. not estimated.

### Statistical dose response analysis

Statistical dose-response regression analyses for the single substances and mixtures were conducted by using a best-fit approach [17]. Various non-linear regression models (logit, probit weibull, generalized logit I and II), which all describe monotonic sigmoid dose-response relationships, were fitted independently to the same data set and the best fitting model was selected on the basis of a statistical goodness-of-fit criterion. Data analysis was performed on pooled data from all the repeat studies. To account for the intra- and inter-study variability associated with this nested data scenario, the generalized non-linear mixed modelling approach was used, in which both fixed and random effects are permitted to have a non-linear relationship with the effect endpoint [19]. For the normalised read-outs (cell number), two sources for random effects were identified: First, the dose-response data for the same chemical from different studies varied in their curve steepness, which was dealt with by including an additional random effect in the steepness model parameter. Secondly, slight shifts of the entire curves based on the log10-transformed concentration scale were observed, which was accounted for by including an additional shift parameter as random effect in the non-linear regression model. The random effects were assumed to follow a Normal distribution. Statistical uncertainties for the estimated effect doses were expressed as 95% confidence belts and approximately determined by applying the bootstrap method [20].

### Calculation of mixture effect predictions using dose addition

The mixture experiments were designed according to the fixed-ratio mixture design, i.e. serial dilutions of a stock solution of a mixture with known mixture ratio were made and then tested against the corresponding DA predictions. The mathematical and statistical procedures used for calculating dose-additive mixture effects according to Equation 1 are described in [7]. The statistical uncertainty for the mixture effects predicted by DA was determined using the bootstrap method [20] and expressed as 95% confidence limits for the predicted mean estimate. Differences between predicted and observed effect doses were deemed statistically significant when the 95% confidence belts of the prediction did not overlap with those of the experimentally observed mixture effects.

### Description of the toxic unit extrapolation approach for a fixed-ratio design

**Figure 2:**
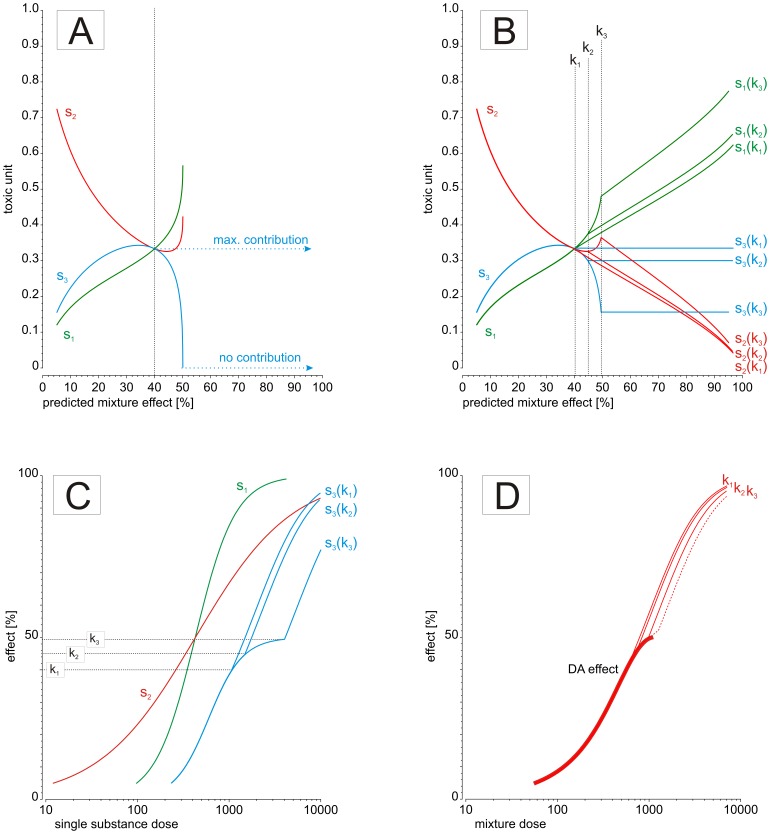
Description of the toxic unit extrapolation method, demonstrated at the hypothetical 3-component mixture from **Figure 1:** (A) toxic units of three compounds (s_1_, s_2_ and s_3_) for mixture effect predictions according to DA. The third compound has a maximum effect of 50% (see Figure 1), thus predictions and toxic units above 50% cannot be calculated. (B) The toxic unit of the third compound is held fixed at effect prediction of and above 49.5%, 45% and 40% (blue line), i.e. s_3_(k_1_), s_3_(k_2_) and s_3_(k_3_), respectively. The corresponding toxic units for the first and second compound are shown as green and red lines, respectively. (C) Dose-response curves of the single compounds after the toxic unit extrapolation, for three different fixed toxic units of the third compound at effect levels k_1_, k_2_ and k_3_. (D) Prediction curve for mixture effects according to DA (solid line), prediction curves of the toxic unit extrapolation assuming maximal toxic unit contributions of the third compound as outlined in B and D (small lines) and assuming no contribution after 50% (dashed line). For details, see Materials and Methods.

For dose ranges that are higher than the effect doses that correspond to the leveling-off range of a partial agonist, the toxic unit extrapolation approach supposes that partial agonists contribute to the total mixture effect by a certain toxic unit. It can be assumed that the value of this toxic unit will vary with total mixture dose and will be different for different partial agonists present in the mixture. It is not immediately obvious which numerical values such toxic units should take, but it is reasonable to suppose that the range of values lies between two extremes, as follows: Firstly, it can be assumed that a partial agonist makes no further contribution to the overall mixture effect when its dose in the mixture approaches its individual saturation range. By using the example sketched out in Figure 1, we have illustrated this scenario in Figure 2 a. Here, toxic units for each of the three components s_1_, s_2_ and s_3_ are plotted as a function of the overall mixture effect. In this example, we have assumed that the three substances are combined at a mixture ratio proportional to the dose of each individual component that produces a 40% effect. It can be seen that the toxic units for each component vary according to the total predicted mixture effect. This is a reflection of the different steepness of each compounds' individual dose response curve (see Figure 1 and equation 1). Under the additivity assumption of DA, the sum of the toxic units corresponding to a specific predicted combination effect (along the vertical lines in Figure 2 a) must equal 1. Of special interest is the point where s_1_, s_2_ and s_3_ have the same toxic unit of 0.333, i.e. where the three toxic unit curves in Figure 2 a intersect. Because the mixture ratio was set proportional to each compounds' ED_40_, this point corresponds to a mixture effect of 40%. As component s_3_ individually produces a maximal effect of only 50% (see Figure 1), its toxic unit curve rapidly approaches zero as the total mixture effect nears 50%. This is because the denominator of the toxic unit term, i.e. the dose of the single compound that elicits an effect equal to that of the mixture, will tend towards infinity as the total mixture effect approaches the effect corresponding to the saturation doses of the partial agonist (here: 50%). To meet the demand of the additivity assumption of DA (i.e. the sum of toxic units equals 1), the curves for the other two components must increase steeply as the total mixture effects comes within reach of 50% (see Figure 2 a).

A second assumption can be made to mark the other extreme of the theoretically possible range of toxic unit values for a partial agonist at mixture doses that exceed its saturation range. Here, its toxic unit is assumed to be fixed to a certain value corresponding to its saturation range (maximum toxic unit assumption). Calculations of predicted mixture effects under the maximum toxic unit assumption require decisions about the numerical value of a partial agonist's toxic unit. We have dealt with this problem in terms of setting the toxic unit for a partial agonist to predefined values corresponding to doses producing certain fractions of the maximal effect level, for example 80%, 90% or 99%. With 50% as the maximal effect, as in our example, this translates into 40%, 45% and 49.5% on the predicted mixture effect scale (see Figure 2 b). The corresponding toxic units for the partial agonist s_3_ can now be read off the graph (Figure 2 b) by seeking the intersections of the three vertical lines k_1_, k_2_ and k_3_ with the toxic unit curve for s_3_, i.e. 0.333 (for 40% mixture effect), 0.29 (for 45% mixture effect) and 0.15 (for 49.5% mixture effect). These values represent three choices for the maximal contribution of s_3_ to the overall mixture effect beyond its own saturation level. The choice of a particular maximum toxic unit for the partial agonist restricts the range of toxic unit values that the remaining two mixture components can assume (the sum of toxic units must still be 1). If, for example, the toxic unit for the partial agonist s_3_ is set fixed to 0.333 for all predicted mixture effects from 40% to 100%, then the sum of toxic units left to be allocated to s_1_ and s_2_ cannot be greater than 0.666. This value is subsequently distributed between s_1_ and s_2_ according to equation 1. The differences between the toxic unit curves for s_1_ and s_2_ reflect the differences in the steepness of the dose-response curves for s_1_ and s_2_ at high effect levels (see Figure 1A). The contribution of compounds with a comparatively shallow dose-response curve (in our case s_2_) to the predicted mixture effect usually decreases with increasing mixture doses, except where all mixture compounds have equally shallow dose-response curves.

Figure 2 c shows the dose-response curve for the partial agonist s_3_ together with the extrapolated curves that result according to the minimal toxic unit assumption and the various fixed values under the maximum toxic unit assumption. These extrapolations are used to calculate the expected additive effects according to DA beyond the leveling-off range of s_3_. The extrapolated curves for s_3_ are not all smooth, but the one corresponding to the highest toxic unit contribution and the smallest fraction of the maximal effect, k_1_ and 80%, respectively, gives the smoothest continuation of s_3_ dose-response curve. In deriving the maximal toxic unit assumption from the experimental data with the 21-component mixture of estrogenic chemicals, we have fixed the toxic unit to that corresponding to 70% of the saturation effect of each partial agonist. The choice of this value was guided by effect levels at which the highest steepness of the non-linear regression functions was observed: for symmetrical functions (e.g., logit) it is half of their maximal model asymptote (50%), whereas for asymmetrical models (e.g., Generalized logit) it was up to 70% of their maximal effect plateau. We performed simulation studies to investigate the optimal cut-off level, and unless the least efficacious compound dominated the mixture composition, we found the value of 70% to be the best trade-off between safeguarding that the toxic unit contribution was really maximal and a sufficient response range used for the prediction by DA. Therefore we recommend this value as default for all data situations where the saturation effect has been estimated with high confidence.

Finally, the range of DA prediction curves derived from the minimum and the various maximum toxic unit assumptions is shown in (Figure 2 d). The bold line represents the anticipated combination effects according to the conventional approach (as in Figure 1), and the thin lines show the predicted effects resulting from the maximum toxic unit assumptions for the three cut-off values above. The thin dashed line depicts the predicted combination effects according to the minimum toxic unit assumption. Although the three different maximum worst-case settings resulted in very different dose-response curves for the third component (Figure 2C), the differences in the prediction curves are rather small. Moreover, the range of predicted mixture effects between the minimum and most conservative maximum toxic unit assumption is small. The leveling-off range for the mixture dose-response curve cannot be calculated using our toxic unit extrapolation approach.

### Mathematical treatment of the toxic unit extrapolation approach

Mathematically, the approach can be described as follows: for simplicity's sake, suppose that only the *n^th^* component of the *n*-compound mixture is a partial agonist. Suppose further that the experimental design for the mixture study is the ‘ray design.’ A ray is defined by a fixed mixing ratio of the components in a specified mixture. Depending on whether the dose *d_n_* of the *n^th^* component in the mixture exceeds the cut-off dose *D_n_* along a fixed ratio ray, dose additivity can be described according to the terminology of Equation 1 as
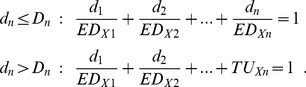
(2)


Here, the toxic unit *TU_Xn_* of the partial agonist is set to a fixed value, with
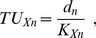
(3)where K_Xn_ is the dose that is extrapolated to the effect level *X*. The limitation to the fixed-ratio design guarantees a unique solution (assuming that only monotonic dose-response relationships are considered). The cut-off dose *D_n_* was selected in our study as dose that produces 70% of the saturation effect. Assuming that the dose-response relationship of the partial agonist can be described by a monotonic function F_n_ and the maximal effect level by a parameter estimate 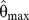 [17], the cut-off dose *D_n_* can then be estimated through the inverse function 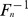 as
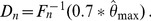
(4)


Consequently, an effect level of x = 0.7* 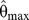 is set as borderline for calculating dose additive response according to DA (Equation 1).

### Adaptation of the Generalised Concentration Addition (GCA) model

We have generalised the GCA model as originally described by [14] for a mixture composed of *n* components, as follows: Let 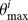 and *ED50_i_* be the Hill model parameters for the i^th^ component describing the maximal effect level and the median effect dose, respectively. The Hill function with slope parameter 1 then describes the response E at a given dose d_1_ as
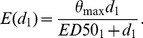
(5)


According to [14] (Equation 17), the combined effect for a binary mixture at given total mixture dose *d_mixture_* can then be described as
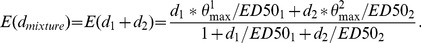
(6)


Assuming that the individual doses of all components as present in the mixture can be expressed as fractions *p* of the total mixture dose *d_mixture_*, with d_i_ = p_i_*d_mixture_ (fixed-ratio mixture design), the dose-additive mixture effect can be calculated for a n-component mixture as
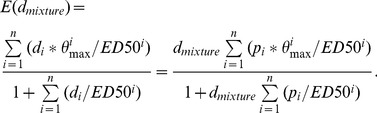
(7)


Increasing mixture doses to infinity, we get the maximal effect level E_max_:
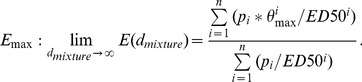
(8)


## Results

**Figure 3:**
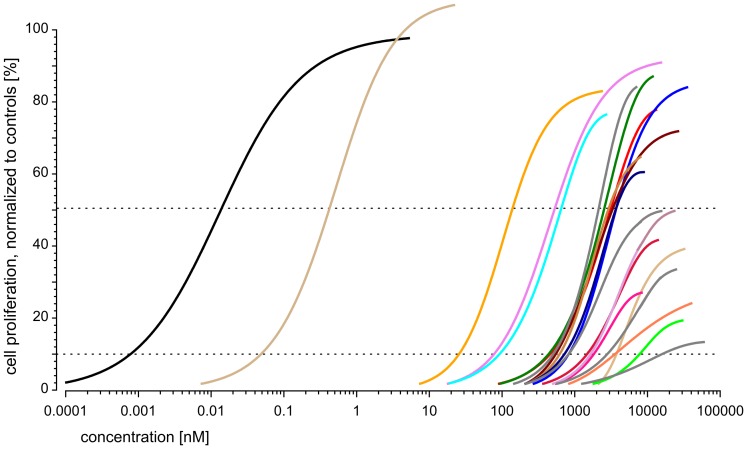
Concentration-response curves for the 21 tested estrogenic chemicals with regression lines derived from the best fitting models for E-Screen *in vitro* data. All agents were tested in at least four independent experiments, run on up to three micro-titer plates, with each plate containing eight increasing concentrations of the test chemical in duplicates (data not shown).

We conducted extensive concentration-response analyses with all the 21 individual chemicals that were included in the mixture. In the interest of providing unbiased response curves for the calculation of expected mixture responses, we used a variety of regression models and selected the best-fitting model for each chemical [17]. The model parameters and effect concentrations are shown in Table 1; some of the data have been published in [16]. Figure 3 shows the best-fitting regression curves for all 21 test components, covering only the non-toxic concentration ranges that were tested experimentally. Most compounds were tested at higher concentrations, but where proliferation responses began to decline as concentrations increased, the data points were judged as cytotoxic and were not included in constructing concentration-response models. The concentration-response curves of the 21 chemicals showed differences in terms of shape, gradient and position, with 17β-estradiol the most potent component (EC10 = 0.00076 nM) and the synthetic musk galaxolide the weakest (EC10 = 15.1 µM). Differences in maximal effects ranged from 100% for the steroids to only 14% for galaxolide. A further 8 chemicals showed maximal effects ranging from 20% to approximately 50% proliferation.

**Figure 4:**
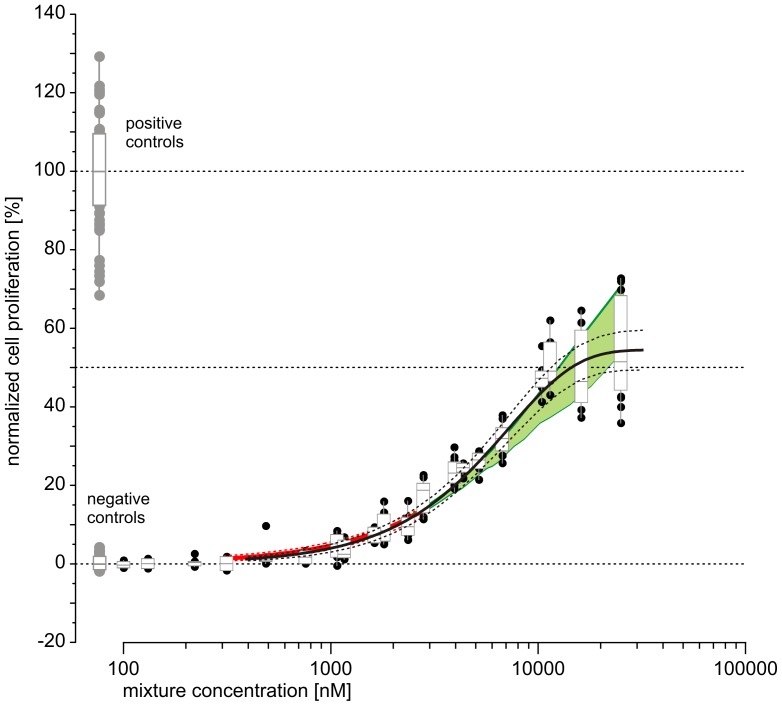
Predicted and observed estrogenic activity of a mixture of 21 components combined according to their individual EC_10_. Observed mixture effects (black circles), controls (gray circles) and regression curve (solid black line, with 95% confidence belt as dotted black lines) are from four independent experiments, each tested on three micro-titer plates. Effect variation is expressed by box and whisker diagrams; the boxes show 1.5 interquartile ranges around the median. Predicted effects were calculated using the DA concept (solid red line), dotted red lines show the corresponding approximate 95% confidence belt. The green lines and the green shaded area are the lower and upper estimates of predictions that are based on toxic unit extrapolations (see Material & Methods for details)

All chemicals were mixed at a ratio proportional to their individual EC_10_ values. We first predicted the combination effects of the 21 chemicals by using the conventional DA concept (equation 1). As expected, this yielded a curve for responses of up to 14%, the maximal effect of the least efficacious component in the mixture, galaxolide (Figure 4, red curve). Due to the large number of repeats in the concentration-response data that formed the basis of the regression analysis of the single components, we were able to produce a DA prediction curve of very low statistical uncertainty (dotted red lines in Figure 4). The width of the 95% confidence belt of the prediction never exceeded 2% on the effect scale.

By using the toxic unit extrapolation approach, it was possible to extend the effect range of the prediction from 14% to between approximately 50% (minimum toxic unit assumption) and 70% (maximum toxic unit assumption) of those seen with the positive control (saturating concentrations of 17β-estradiol) (Figure 4). For the extrapolation of mixture effects according to the maximum toxic unit assumption, we fixed the toxic unit of each single partial agonists to a value associated with 70% of its maximal effect plateau, as estimated from the regression models (Table 1). For extrapolations derived from the minimum toxic unit assumption, toxic units were set to zero (Equation 3).

The predictions were then tested experimentally. The experimental data showed comparatively little variation in the low concentration range, but variation increased as the mixture effect curve approached its plateau with a maximal cell proliferation of approximately 55%.

Over the entire range of observations, the predicted combination effects agreed very well with the experimentally observed data (Figure 4), both for the predictions derived from Equation 1 (red curve) and for those from the toxic unit extrapolation approaches (green curves). For a total mixture concentration of 10,000 nM, the DA predictions according to the minimum and maximum toxic unit assumptions spanned responses of between 35% and 45% proliferation, respectively. The experimentally observed effects generally agreed better with the extrapolation derived from the maximum toxic unit assumption.

**Figure 5:**
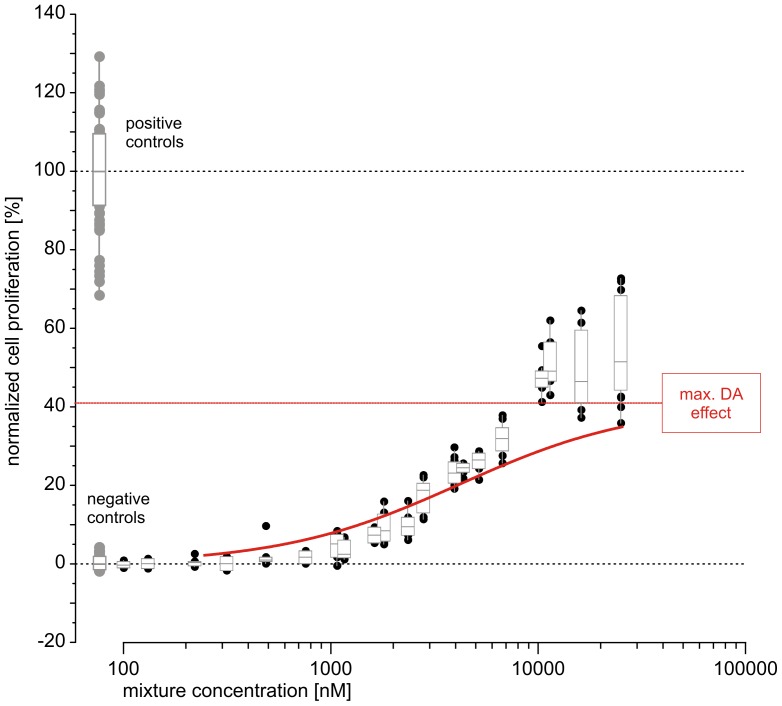
Predicted and observed estrogenic activity of the mixture of 21 components shown in Figure 4. The predicted effects were calculated using the model of DA (solid red line) according to the GCA approach by [14]. Observed mixture effects (black circles) and controls (gray circles) are from four independent experiments, each tested on three micro-titer plates. Effect variation is expressed by box and whisker diagrams; the boxes show 1.5 interquartile ranges around the median.

Figure 5 shows a comparison of the experimental mixture data with the prediction derived from the GCA approach based on Hill functions for all components' individual dose-response data. Here, the prediction curve from the toxic unit extrapolation approach is not included. At low concentrations, the GCA overestimated the observed responses, but at higher concentrations the GCA model underestimated the proliferative effects of the mixture. The model predicted a maximal response of 41%. Had we used our toxic unit extrapolation approach based only on Hill functions (with steepness model parameters fixed to 1), and not with the variety of regression models shown above, we would have obtained a curve very similar to the one shown in Figure 5, with its overestimation of combination effects in the low concentration range (data not shown). This highlights the importance of accurate descriptions of dose-response relationships for each mixture component for achieving valid mixture effect predictions.

## Discussion

We show that the toxic unit extrapolation approach is able to produce DA predictions that agree very well with our experimentally observed data. The approach affords sufficient flexibility for dealing with mixture components that exhibit concentration-response relationships with varied slopes, positions and maximal effects. Our findings help to dispel the widespread misconception that DA is only applicable to mixtures composed of chemicals that show parallel response curves. However, the general formulation of DA neither assumes a specific shape of each concentration-response curve of the components, nor a specific relationship between the curves. The general application of DA is sometimes confused with “simple similar action”, a special case of DA which requires that the individual curves of the components are dose-parallel, as in the “toxic equivalence factor” (TEF) concept. Dose-parallel curves occur with endpoints relatively close to molecular events, such as receptor binding or receptor activation, but often do not appear effects representative of higher levels of biological organisation. The lack of parallelity with apical endpoints may be explained in terms of differences in the toxicokinetic and toxicodynamic behaviour of substances. Such phenomena are relevant to the E-Screen assay where differential uptake, metabolism and transport out of the cells may lead to non-parallel curves. Furthermore, the E-Screen is more integrative assay than e.g. an ER binding assay, and responds not only to the classical activation of the ER by direct binding of the ligand, but also to indirect activation, such as phosphorylation by protein kinases.

We noticed that the predictions derived from the minimum toxic unit assumption, where partial agonists are presumed to contribute nothing to the overall mixture effect at doses beyond their saturation ranges, did not perform as well as the maximum toxic unit assumption. This may be a reflection of biological realities: Partial agonists will still make a contribution to the overall mixture effect when their doses fall in their leveling-off range, a phenomenon that the minimum toxic unit assumption is by definition unable to capture. Even so, it is striking that the prediction differences between the two extremes of extrapolations were not very large, particularly not in the range of mixture doses up to the median effect level of the mixture response curve.

If the maximum toxic unit assumption is better suited to deal with mixture scenarios that require flexible regression models for describing accurately dose-response relationships, it is all the more important to consider the implications in terms of data requirements and data quality. As detailed during the description of the toxic unit extrapolation approach, the choice of the highest effect dose for each partial agonist component that can be considered acceptable for DA predictions is crucial. In our study we consistently used the same cut-off criterion, i.e. 70% of the maximum effect level. Thus, the quality of prediction strongly depends on accurate estimations of the saturating effect levels for each compound. In regression analysis, this approximation is achieved by estimating the maximum asymptotic model parameter (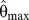 in Table 1), but this requires effect data of sufficient quality. With the E-Screen assay this may sometimes be problematic, for two reasons: firstly, between-study data variation is highest for high effect levels, i.e. more data is needed to achieve effect estimates for higher concentrations that are statistically comparable with low effect estimates. Secondly, at concentrations nearing the saturation range cytotoxicity may become prominent, and this may result in a down-turn of effects, such that the maximal effect plateau occurs only in a narrow range of concentrations. This phenomenon has been observed with certain estrogenic chemicals [9]. In such situations, the quality of estimating saturating effect levels strongly depends on the spacing of the tested concentrations, which has to be done judiciously. However, these complications were not relevant with the estrogenic chemicals included in our mixture.

The alternative CGA model did not produce mixture effect predictions of high accuracy and proved to be inferior to our toxic unit extrapolation approach. To a very large degree, this was due to the problems we encountered in using the Hill function as the regression model for the single components in our mixture. We observed that the Hill function generally produced regression models with poorer goodness of fit than the regression models selected by the best-fit approach [17]. The Hill function generally overestimated the single component's effects at low concentrations (data not shown). Perhaps due to the fact that it has a symmetrical shape, it lacks the flexibility to accurately approximate the effects of the tested chemicals, particularly in the low dose range. This may explain why the CGA model, which relies on the Hill function with slope parameter 1, consistently overestimated the observed mixture responses at low effects. Mathematically, DA is an averaging concept, with the predicted effect doses corresponding to the weighted harmonic mean of all individual effect doses, where the weights relate to the fractions of the individual compounds in the mixture. Therefore, if the regression model for the single components always produces systematic errors in the same direction, the DA prediction will be biased in the same way, as was indeed observed with the CGA model (Figure 5). The advantage of the CGA method is in its comparative ease of use, with less demanding calculations. This method will therefore be appropriate when the mixture components exhibit dose-response curves with similar gradients, or when less accurate predictions are judged to be sufficient.

The mixture of 21 estrogenic chemicals showed proliferation responses that were matched accurately by the prediction according to DA and, at higher effect concentrations, according to the toxic unit extrapolation method. These outcomes confirm those from previous studies ([7]; [16]), where DA approximated well the observed response from various multi-component mixtures of estrogenic agents, suggesting that for cell proliferation and testing conditions defined by the E-Screen assay the pharmacological requirements of “similar action” of the DA concept were fulfilled. We applied the DA extrapolation approach also successfully to multi-component mixture studies in various other *in vitro* testing systems, e.g. on reporter-gene endpoints for estrogenicity (ERLUX) [21] and anti-androgenicity (MDA-kb2) [22]. Experimental evidence also suggests that potential pharmacokinetic or dynamic “interactions” between compounds were not pronounced enough to become detectable which would have diminished the predictive power of DA. Considering that the E-SCREEN represents the highest level of biological complexity of all the *in vitro* assays in use for the screening of endocrine active chemicals and taking into account the large numbers of chemicals with varied structural features that were tested in combination, we conclude that DA provides reasonable approximations for combination effects of estrogenic chemicals with the endpoint of cell proliferation. In addition, our study has demonstrated that the toxic unit extrapolation method is capable of extending the applicability of DA to combinations of agents with differing saturating effects. The E-Screen is considered as a biomarker for internal EDC exposure and the toxic unit prediction tool developed here will be useful to estimate the combined internal effective doses in humans on the basis of chemical analytical data of tissue levels of multiple estrogenic agents. Estimations of combined effects can now include compounds that exhibit much lower maximal effect responses than the reference compound estradiol. Our new approach thus overcomes a limitation which has previously hampered the estimation of cumulative internal EDC exposures. The new method can also be applied to other classes of environmental pollutants, with other effect profiles.
